# A New Relevant Integrated Radiologic and Surgical Classification Scheme for Giant Cell Tumors of Bones

**DOI:** 10.5435/JAAOSGlobal-D-24-00321

**Published:** 2025-07-17

**Authors:** Yu-Cherng Chang, Juan Pretell-Mazzini, H. Thomas Temple, Roxana Soler, Julian Purrinos, Andrew E. Rosenberg, Emily Jonczak, Ty K. Subhawong

**Affiliations:** From the Department of Radiology, Jackson Memorial Hospital, Miami, FL (Dr. Chang); the Baptist Heath South Florida, Plantation, FL (Dr. Pretell-Mazzini); the Department of Orthopaedics, Leonard M. Miller School of Medicine, University of Miami, Miami, FL (Dr. Temple); the Nova Southeastern School of Allopathic Medicine, Davis, FL (Dr. Soler); the Leonard M. Miller School of Medicine, University of Miami (Mr. Purrinos); the Department of Pathology and Laboratory Medicine, Leonard M. Miller School of Medicine, University of Miami (Dr. Rosenberg); Department of Medicine, Division of Hematology and Oncology, Leonard M. Miller School of Medicine, University of Miami (Dr. Jonczak); and the Musculoskeletal Radiology Division, Department of Radiology, Leonard M. Miller School of Medicine, University of Miami, Miami, FL (Dr. Subhawong).

## Abstract

**Introduction:**

Conventional classification systems for giant cell tumors (GCTs) lack robust correlation with management and clinical outcomes. We propose a new radiologic classification system based on surgically relevant features to address this shortcoming.

**Methods:**

This IRB-approved single-institution retrospective study involved 35 extremity GCTs from 2013 to 2023 with preoperative radiographs and cross-sectional imaging (MRI and/or CT). An experienced musculoskeletal (MSK) radiologist and orthopaedic oncologist independently assessed tumors according to the Campanacci or new grading system, defined on 1 to 3 scale: (1) intraosseous contained tumor, (2) intraosseous noncontained tumor with extraosseous implant accessible through single incision, and (3) intraosseous noncontained tumor with an extraosseous soft tissue implant nonaccessible from single incision alone. Interrater agreement was determined through the intraclass correlation coefficient. The two-way Friedman test with rater and grading system as factors was used to compare system grading similarity.

**Results:**

Thirty patients underwent curettage, five underwent resection; 10 experienced local recurrence. Intraclass correlation coefficients between raters for the Campanacci and novel grading systems were 0.83 and 0.79, respectively. However, compared with the novel system, Campanacci grades were significantly higher by an average of 0.34 ± 0.68 and 0.46 ± 0.70 for the first and second raters, respectively (*P* = 0.003). None of the patients who underwent resection experienced local recurrence, but in patients who underwent curettage, recurrence rates were higher in Campanacci versus novel grade 1 tumors (29% vs. 17%).

**Discussion:**

The novel GCT grading system demonstrates excellent interrater agreement, and classified more nonrecurrent curetted tumors as low grade, suggesting improved predictive performance compared with the Campanacci classification.

Giant cell tumors (GCTs) of bone are locally aggressive, rarely metastasizing primary bone tumors, with an estimated incidence of 1.03 to 1.66 per million.^1^ They most often affect adults in the 3rd and 4th decades, occurring predominantly in the metaphyseal-epiphyseal regions of long bones, particularly around the knee.^2^ Symptoms at presentation include pain, tenderness, swelling, diminished joint motion, and less frequently pathologic fractures,^3^ although metastases develop in approximately 5% of patients,^4^ there are no reliable histologic or radiologic predictors of malignant behavior.

Surgical management remains the cornerstone of treatment, with conservative approaches, for example, intralesional curettage with adjuvant treatment, favored to preserve function. When combined with local adjuvants such as phenol or hydrogen peroxide, recurrence rates are reported around 15%.^5^ However, curettage is linked to a greater risk of recurrence compared with wide resection.^6-8^ Consequently, cases presenting with increased risk factors, including soft tissue extension or pathologic fractures, often require wide resection to mitigate the risk of recurrence effectively.^9^ In light of these tradeoffs, the choice between curettage and resection needs to be weighed carefully for each patient, and determining GCT of bone biologic aggressiveness influences that decision process.

Various classification systems for GCTs have been proposed over multiple decades (Table 1). Jaffe et al,^10^ in 1940, initially classified GCTs as benign, aggressive, and malignant based on histologic appearance. This was expanded upon in 1975 by Campanacci et al, who introduced a radiographic grading system that categorized lesions into three grades based on tumor margins and cortical involvement.^11,12^ In 1977, d'Aubigne and Tomeno introduced a classification system that divided GCTs into slow versus aggressive tumors, adding clinical information, relating clinical course and local inflammatory response, to similar radiographic criteria.^13^ Enneking et al,^14^ in 1980, proposed a pathoradiologic staging system for benign bone tumors, including GCT, with pathology based on Broder classification and incorporation of local extension versus metastases.

**Table 1:** Historical Giant Cell Tumor Classification Schemes and Descriptions

Classification System	Slow/Low Grade/Grade 1	Medium Grade/Grade 2	Aggressive/High Grade/Grade 3
Jaffe et al,^10^ (histopathologic)	Giant cells without mononuclear cells or mitotic activity	Mononuclear stromal cells present with moderate atypia and mitotic activity	Giant cells with atypia and pleomorphism with mitotic activity
Campanacci et al,^12^ (radiographic)	Well-defined margin and intact cortex	Relatively well-defined margin without a radiopaque rim and a thinned, moderately expanded cortex	Indistinct borders and clear cortical destruction
D'Aubigne and Tomeno,^13^ (clinical/radiographic)	*Clinical*: Long time since onset of symptoms (1-2 years), long time between treatment and recurrence (3-5 years), no local inflammation *Radiographic*: Sharp margins, preserved cortex, slow extension of defect	—	*Clinical*: Short time since onset of symptoms (∼6 months), short time between treatment and recurrence (∼6 months), local inflammation may be present *Radiographic*: Blurred margins, destroyed cortex, rapid extension of defect
Enneking et al,^14^ (histopathology/radiologic)	1A: *Pathology*: <50% undifferentiated cells *Radiologic*: Intracompartmental (local) extension, no metastasis 1B: *Pathology*: <50% undifferentiated cells *Radiologic*: Extracompartmental extension, no metastasis	2A: *Pathology*: ≥50% undifferentiated cells *Radiologic*: Intracompartmental (local) extension, no metastasis 2B: *Pathology*: ≥50% undifferentiated cells *Radiologic*: Extracompartmental extension, no metastasis	*Pathology*: Any amount of undifferentiated cells *Radiologic*: Metastasis with any extension

In clinical practice, the Campanacci system is most often used due to its simplicity and relatively straightforward management implications, where grade 1 and 2 lesions are amenable to intralesional curettage, and grade 3 lesions may require wide resection.^15^ Nevertheless, the Campanacci system has never demonstrated a definite correlation with prognosis. The literature is mixed with some studies showing no correlation,^16-19^ including Campanacci et al,^11^ raising questions about reliance on a grading system that poorly predicts clinical outcomes. In light of these shortcomings, we believe that GCT of bone classification can be improved by adding consideration of cross-sectional imaging, which is now routinely obtained for surgical planning, and by aligning radiologic classification with features having surgical implications.

We developed a novel integrated radiologic and surgical classification scheme for GCTs of bone, based on whether the tumor is entirely contained within cortical margins, violates cortex but remains accessible from a single incision, or contains extraosseous implants that would necessitate two incisions for surgical intervention to local control without doing a wide local excision. We tested this novel classification scheme against the Campanacci grade and determined interreader reliability, with the aim of developing a more clinically applicable framework for surgically treating GCTs of bones.

## Methods

As an IRB-approved, retrospective study, requirement for informed consent was waived. Patients were identified from a review of GCT of bone cases at our institution over a 10-year period from 2013 to 2023. Exclusion criteria included any patient without a histologically confirmed diagnosis of GCT or lack of pretreatment radiograph and contemporaneous cross-sectional imaging (MR and/or CT within 2 months). Recurrent or metastatic tumors were also excluded.

**Figure 1:**
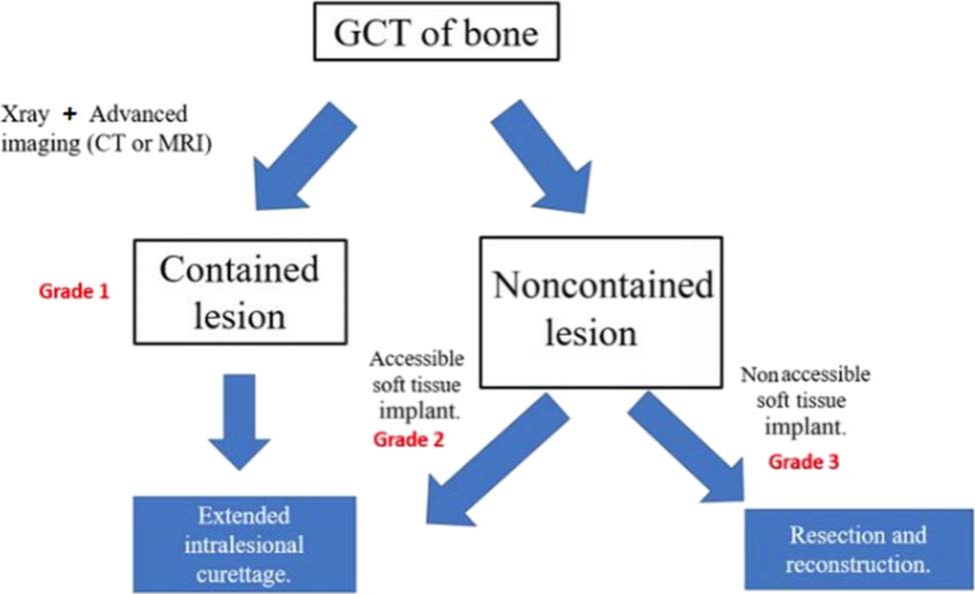
Flow diagram details the proposed novel classification system.

**Figure 2:**
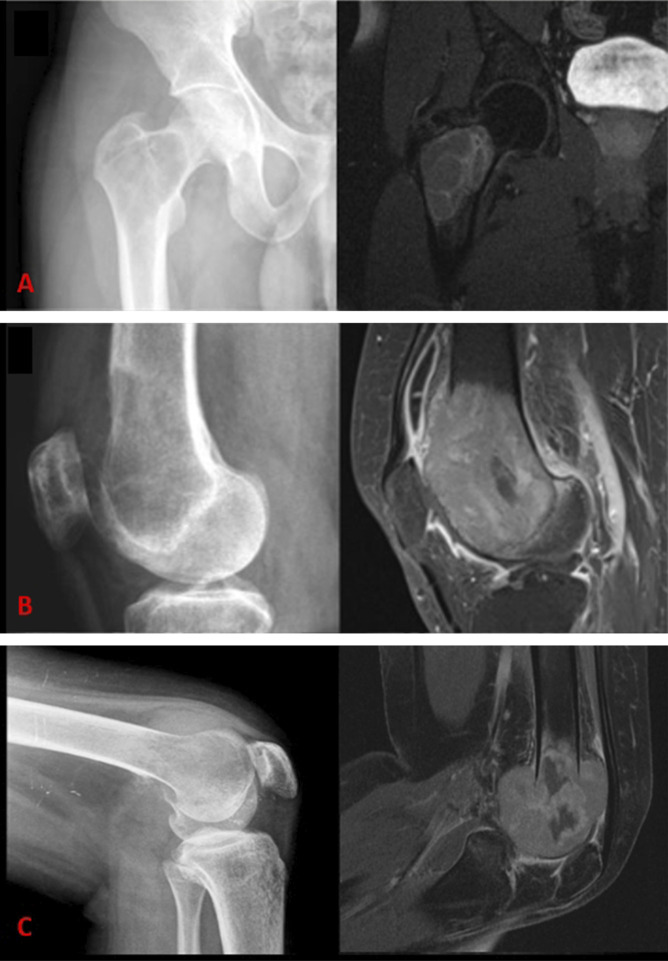
Representative examples are shown of novel classification system (**A**) grade 1, (**B**) grade 2, and (**C**) grade 3 GCTs of bone with paired radiographs and cross-sectional imaging.

An experienced MSK radiologist and orthopaedic oncologist independently assessed the tumors according to the Campanacci or new grading system (Figure 1), consisting of a 1 to 3 scale: (1) intraosseous contained lesion, (2) intraosseous noncontained tumor with extraosseous implant accessible through single incision, and (3) intraosseous noncontained tumor with an extraosseous soft tissue implant nonaccessible from single incision alone. Extraosseous soft tissue implants requiring multiple incisions are defined as those in separate, distinct compartments (dorsal and volar, or medial and lateral sides of long bone, etc). Representative examples are shown in Figure 2, A–C. Cross-sectional imaging protocols varied among our institution and various outside hospitals (4 from home institution and 31 from outside hospitals) with both contrast-enhanced and noncontrast CTs, and 1.5 and 3.0 T MRIs used (32 patients with MRIs and 4 with CTs). In general, multiple planes and sequences were reviewed to allow optimal characterization of tumor extension, but generally axial sequences perpendicular to the long bones were found to be most beneficial.

## Statistical Analysis

Baseline patient demographics and descriptive tumor characteristics were computed. Interrater agreement was determined through the intraclass correlation coefficient based on a single-rating, absolute-agreement, 2-way random-effects model implemented through a publicly shared library (https://www.mathworks.com/matlabcentral/fileexchange/22099-intraclass-correlation-coefficient-icc) in the Matlab R2022a programming environment (MathWorks). A two-way Friedman test with rater and grading system as factors was used to compare similarity of grades between the two systems in the SPSS Statistics 22 (IBM) software suite.

## Results

Thirty-five patients with GCT (40.2 ± 15.1 years old; 51% male and 49% female) were included with an average follow-up time of 2.6 years. The most common locations for GCTs were in the knee with 60% of cases, followed by 20% in the wrist, 9% in the elbow, and 11% in varied other locations with all of the GCTs present in long bones. Patients underwent curettage in 30 of the cases and wide local resection in the other 5. Ten of the patients experienced local recurrence, all of whom underwent curettage. Nine of the patients received either neoadjuvant or adjuvant denosumab with eight of these patients being part of the recurrent population.

**Figure 3:**
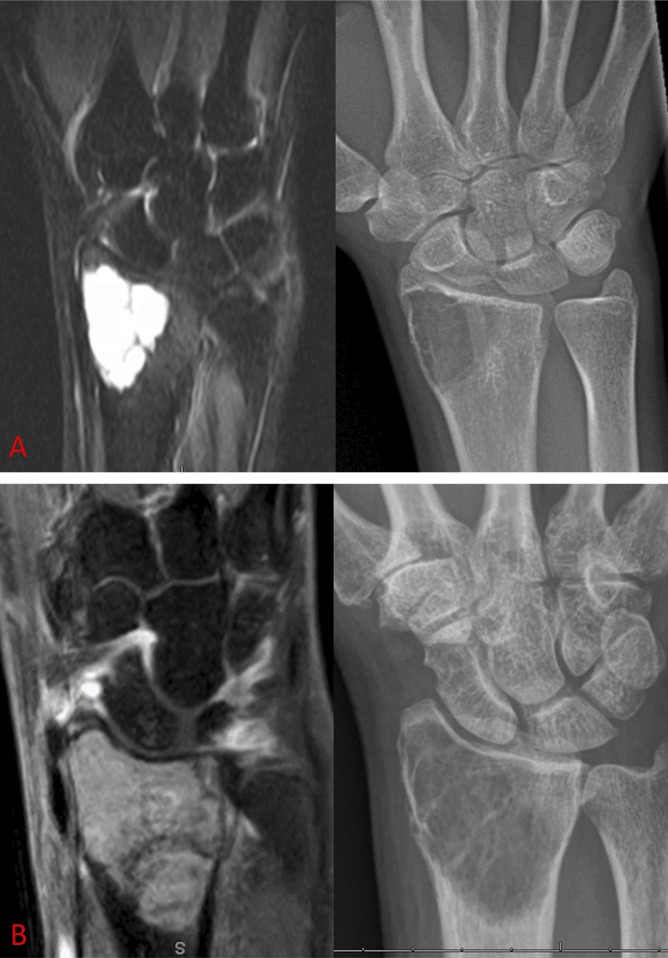
Examples are shown of paired MRIs and radiographs of GCTs of bone, which were downgraded in both raters from (**A**) Campanacci grade 2 to novel grade 1 and (**B**) Campanacci grade 3 to novel grade 1.

**Figure 4:**
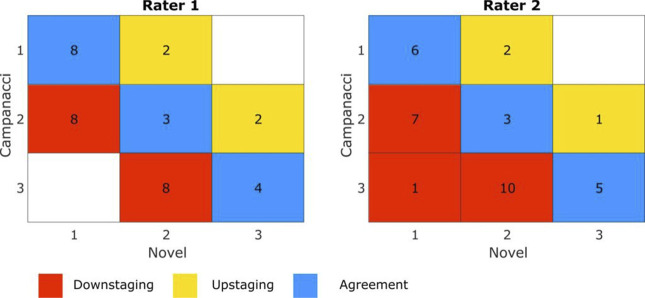
Depicted confusion matrices demonstrate concurrence of Campanacci and proposed the novel classification system for each rater. Boxes corresponding to agreement were highlighted in blue, downstaging in red, and upstaging in yellow. Novel classification grades tended to be lower than Campanacci classification grades in both raters.

ICCs between raters for the Campanacci and novel grading systems were 0.83 and 0.79, respectively. Overall, 10, 13, and 12 tumors were classified Campanacci grades 1, 2, and 3, respectively by rater 1, compared with 8, 9, and 16 for rater 2. 16, 13, and six tumors were classified novel grades 1, 2, and 3, respectively, by rater 1, compared with 14, 15, and six for rater 2. The novel system tended to downgrade tumors for both raters (Figures 3, A and B), Campanacci grades being higher by an average of 0.34 ± 0.68 and 0.46 ± 0.70 for the first and second raters, respectively (Figure 4). This difference in grading between the two systems was statistically significant (*P* = 0.003, Friedman test).

**Figure 5:**
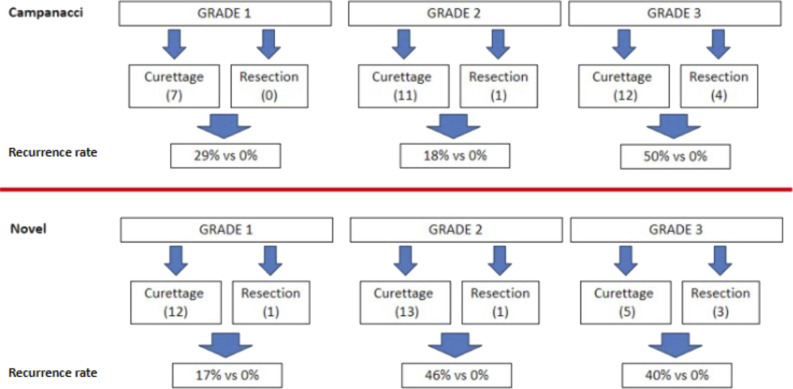
Diagram showing the recurrence rates of delineate between giant cell tumor (GCT) of bone patients receiving curettage or resection, between grading systems, and between degree of severity. Of note, no recurrences occurred in GCT of bone patients who were treated with wide resection. In addition, recurrence rates were higher in grade 1 Campanacci versus novel grading system GCTs.

None of the patients who underwent resection experienced local recurrence; however, in patients who underwent curettage, recurrence rates were higher in Campanacci grade 1 versus novel grade 1 tumors, with 29% (n = 7) of the Campanacci grade 1 tumors recurring versus 17% (n = 13) of the novel grade 1 tumors (Figure 5). Recurrence rates in Campanacci grade 2 tumors were 18% (n = 12) vs. 46% (n = 14) in novel grade 2 tumors. Recurrence rates in Campanacci grade 3 tumors were 50% (n = 16) vs. 40% (n = 8) in novel grade 3 tumors. It also follows that recurrence rates in the combination of novel grade 2 and 3 tumors were higher than in the combination of Campanacci grade 2 and 3 tumors (36% vs. 29%).

## Discussion

Our study introduces a novel grading system for GCTs of bone, incorporating cross-sectional imaging features that affect the surgical approach, differing from the traditionally used Campanacci grading system, which is based solely on radiographic appearance. The novel system demonstrates good interrater reliability, comparable with the Campanacci system, while providing distinct and separate information as evidenced by the notable difference in grades among patients. The novel system frequently downstages tumors compared with the Campanacci classification, and more accurately classified nonrecurrent tumors as low grades.

Implementing the novel grading system is straightforward, based only on imaging features, and thus independent of clinical course or histopathology which may be unknown to the diagnostic radiologist at the time of interpretation. The system does depend on cross-sectional imaging but is independent of the modality, whether MRI or CT, and such imaging is universally obtained as current preoperative standard of care.

Incorporating cross-sectional imaging provides better characterization of GCTs of bone, which can be important to local recurrence. The presence of soft tissue involvement has been suggested to correlate with increased recurrence rates,^20,21^ potentially explaining the greater correlation with recurrence of the novel grading system, which considers soft tissue extension in its criteria, in contrast to the Campanacci system. It follows that the definition of the margins of the tumor, which is used in most GCT classifications, may have less relation to the clinical course of patients than previously thought. Possible explanations have been proposed such as the concept of “invisible margins,” where the highest rate of tumor growth is through cancellous bone, leading to a radiographically occult appearance, or the presence of small foci of aggressive margins incongruent with the overall appearance of tumor margins.^22^ Given the novel grading system is based on surgical features, the system can better inform of the status of GCTs of bone as it pertains to the surgical approach.

Despite these promising findings, our study is not without limitations. GCTs are relatively uncommon, and we were limited to 35 patients at a single institution after accounting for our exclusion criteria (patients without imaging in picture archiving and communication system (PACS), with recurrent or metastatic GCT on presentation, etc). Only two raters may have been involved with cases during routine clinical care, giving rise to potential for recall bias in recognizing cases and their oncologic outcomes. However, the findings comparing interrater reliability and differences within grading systems were well within statistical significance (*P* < 0.01). Although a larger data set and more raters would be desirable in increasing confidence and included as part of future plans, the direction of findings would likely remain the same.

In confounding variables, the heterogeneity of available cross-sectional imaging (eg, MRI or CT, contrast or noncontrast, and differences in sequences or phases) could influence reliability among raters. The overwhelming majority of patients in this study had MRIs accompanying their radiographs (32 patients with MRIs and 4 with CTs), which somewhat limits exposure to this confounder although there were certainly differences in protocol, contrast administration, and MRI machines. It could become a greater issue when generalizing to other practices, where the variation in available imaging will be even greater. In addition, it should be noted that eight of the patients received denosumab at some point in their clinical course, either before or after surgical treatment. The use of denosumab has recently been suggested to increase the risk of local recurrence after local curettage,^23,24^ although indication bias (denosumab therapy may downstage more aggressive tumors so that they are amenable to curettage) is a potential confounder, and the role of long-term suppressive therapy remains unknown.

Finally, it should also be noted that there was no expected increasing trend in recurrence rate between GCTs of bone rated grades 2 and 3 with the novel grading system. Although counterintuitive, this suggests the presence of soft tissue extension raises the local recurrence risk to a fixed extent irrespective of the accessibility of the tumor from one or two incisions; larger studies with recurring grade 2 and 3 tumors are needed to address this possibility. Nevertheless, recurrent tumors were more frequently classified as higher grade (grades 2 and 3) on the whole when comparing the novel and Campanacci grading systems, indicating a benefit in at least stratifying between grade 1 versus grades 2 and 3 with the novel system. Future larger studies may also be able to improve on recurrence prediction by exploring the addition of other features to further stratify recurrence rates once tumor grades are greater than 1. Preliminary investigation showed, within novel grade 2 and 3 tumors, that correlation of recurrence with length of extraosseous tumor extension approached significance (*P* = 0.06), although measurements of tumor size (longest axial diameter and product of longest two axial dimensions) did not (*P* = 0.13 and 0.37, respectively).

This study introduces a novel grading system for GCTs of bone that is similar in reliability to the Campanacci grading system while offering greater surgical relevance. Future studies validating the system in larger, multicenter investigations should also explore the relationship of the grading system with clinical correlates, such as local recurrence and metastatic rates, denosumab treatment effect, and patient-reported outcomes. The integration of the system into clinical practice could enhance radiologist communication with surgeons for surgical planning, improve prognosis, and ultimately lead to better outcomes for patients with GCT.
